# Comparative Efficacy and Safety of PD-1/PD-L1 Inhibitors for Patients with Solid Tumors: A Systematic Review and Bayesian Network Meta-analysis

**DOI:** 10.7150/jca.49325

**Published:** 2021-01-01

**Authors:** Qingyuan Huang, Yuzhen Zheng, Zhendong Gao, Lianxiong Yuan, Yihua Sun, Haiquan Chen

**Affiliations:** 1 Department of Thoracic Surgery and State Key Laboratory of Genetic Engineering, Fudan University Shanghai Cancer Center, Shanghai, China; 2 Institute of Thoracic Oncology, Fudan University, Shanghai, China; 3 Department of Oncology, Shanghai Medical College, Fudan University, Shanghai, China; 4 Department of Thoracic Surgery, Affiliated Cancer Hospital & Institute of Guangzhou Medical University, Guangzhou, China; 5 Department of Thoracic Surgery, The Sixth Affiliated Hospital, Sun Yat-sen University, Guangzhou, China; 6 Office of Research Service, Third Affiliation Hospital of Sun Yat-Sen University, Guangzhou, Guangdong, China

## Abstract

**Purpose:** The clinical use of immunotherapies targeting programmed death 1 (PD-1)/programmed death ligand 1 (PD-L1) is rapid expanding, but the equivalency of these inhibitors remains unclear. We aimed to comprehensively compare the efficacy and safety of PD-1/PD-L1 inhibitors with a systematic review and Bayesian network meta-analysis

**Methods:** We searched PubMed, Web of Knowledge, related reviews and abstracts for randomized controlled trials of five PD-1/PD-L1 inhibitors for patients with solid tumors before November 30^th,^ 2018. We estimated summary hazard ratios (HRs) for overall survival (OS) and progression-free survival (PFS), and odds ratios (ORs) for grade 3-5 treatment-related adverse events (TrAEs) using pairwise and network meta-analysis with random-effects. This study was registered with PROSPERO (#CRD42018116624).

**Results:** Totally, 43 reports of 35 trials comprising 21261 patients were eligible for the analysis. Nivolumab, pembrolizumab, atezolizumab and durvalumab were more effective than control treatment, and no significant differences were identified in OS and PFS between any two inhibitors. Avelumab was associated with significantly inferior OS to nivolumab (HR 1.37, 95%CrI 1.05-1.78) and pembrolizumab (HR 1.33, 95%CrI 1.02-1.73), and with inferior PFS to nivolumab (HR 1.60, 95%CrI 1.03-2.51). Compared with placebo, nivolumab had increased risk of grade 3-5 TrAEs (OR 2.35, 95%CrI 1.35-4.17). Compared with standard-of-care, nivolumab (OR 0.39, 95%CrI 0.28-0.54), pembrolizumab (OR 0.43, 95%CrI 0.30-0.60), atezolizumab (OR 0.37, 95%CrI 0.21-0.64) and avelumab (OR 0.24, 95%CrI 0.12-0.48) significantly reduced grade 3-5 TrAEs. There were not significant differences in grade 3-5 TrAEs between any two inhibitors.

**Conclusion:** This Bayesian network meta-analysis revealed that nivolumab, pembrolizumab, atezolizumab and durvalumab yielded equivalent survival, while avelumab was associated with unfavorable survival. PD-1/PD-L1 inhibitors were comparable in the risk of TrAEs, and safer than conventional therapies.

## Introduction

In the past decade, immunotherapies targeting the programmed death 1 (PD-1)/programmed death ligand 1 (PD-L1) pathway have been demonstrated to induce long-lasting survival benefits among patients with a wide spectrum of cancers, and have transformed the treatment paradigm^1^,^2^. To date, the U.S. Food and Drug Administration (FDA) has approved three PD-1 inhibitors (nivolumab, pembrolizumab, and cemiplimab) and three PD-L1 inhibitors (atezolimumab, durvalumab and avelumab) for the treatment of solid tumors in different clinical settings, including non-small-cell lung cancer (NSCLC), melanoma, urothelial carcinoma, head and neck squamous cell carcinoma (HNSCC) and merkel cell carcinoma (MCC). Moreover, nivolumab and pembrolizumab are recommended as the treatment of choice in the first-line setting for melanoma and NSCLC by the National Comprehensive Cancer Network (NCCN) Clinical Practice Guidelines in Oncology (https://www.nccn.org/), as phase 3 randomized trials have proven the superiority over standard of care (SoC). They also provide clinically significant survival advantage among patients with stage III melanoma after complete resection^3^,^4^, and are category 1 options for adjuvant therapy.

Despite the remarkable clinical success of anti-PD-1/PD-L1 immunotherapy, it is unclear whether these inhibitors have the same effects on restoring anti-tumor immunity, resulting in the difficulty in choosing the best inhibitor in the clinic. In the aspect of tumor immune biology, PD-1 inhibits signaling downstream of the T cell receptor to regulate immune cell activity within tissue and tumors, when engaged by its two ligands, PD-L1 and PD-L2^5^. In addition to interacting with PD-1, PD-L1 also interacts with CD80 expressed on T cells and mediates an inhibitory signal^6^. What's more, PD-L1 could function as a receptor to mediate tumor cell-intrinsic signals to affect cell growth in the absence of immunity^7^,^8^. PD-1 inhibitors could block PD-1 from interacting with both PD-L1 and PD-L2, but not the PD-L1/CD80 interaction. By contrast, PD-L1 inhibitors could block the PD-L1/PD-1 and PD-L1/CD80 interactions, but leave PD-L2/PD-1 pathway intact. Additionally, PD-L1 inhibitors could also directly interfere the immune-independent cell growth mediated by PD-L1. Lastly, PD-L1 antibody has the potential to induce antibody-dependent cellular cytotoxicity when binding to the surface of tumor cells, contributing to its clinical activity^9^.

In addition to the differences of the molecular biological functions of PD-1/PD-L1 inhibitors, differences of their performance in clinical trials were noted. The optimal efficacy of pembrolizumab was observed in treated NSCLC patients with PD-L1 expression in at least 50% of tumor cells, revealing the predictive value of PD-L1 expression as a biomarker^10^,^11^. Nevertheless, nivolumab 12 or atezolizumab 13 treatment led to a clinically relevant improvement of survival versus docetaxel in treated NSCLC patients, regardless of PD-L1 expression level. Besides, pembrolizumab monotherapy significantly improved the survival over chemotherapy in treatment-naive advanced NSCLC patients with PD-L1 strong positive expression^14^, while nivolumab monotherapy failed to extend survival in the first-line trial^15^. Collectively, these findings bright up the question whether PD-1/PD-L1 inhibitors have equivalent efficacy and safety.

To the best of our knowledge, there have been not randomized trials published or in progress which “head-to-head” compare these PD-1/PD-L1 inhibitors so far. The lack of robust data evaluating the efficacy and toxicity of these inhibitors is a major dilemma of the clinic practice. Bayesian network meta-analysis of present evidence uses a common comparator to achieve indirect comparison when a head-to-head clinical trial is absent, and combines direct and indirect comparisons to simultaneously compare multiple interventions^16^. Therefore, we performed a systematic review and network meta-analysis of all randomized trials to compare the efficacy and safety of the FDA-approved PD-1/PD-L1 inhibitors for patients with solid tumors.

## Methods

### Study eligibility and selection

We conducted the systematic review in line with the recommendations of the Cochrane Collaboration the Preferred Reporting Items for Systematic Reviews and Meta-Analyses (PRISMA) guidelines. The protocol was prospectively registered with the PROSPERO database (registration ID: CRD42018116624). All randomized controlled trials that comparing the overall survival (OS) or progression-free survival (PFS) of PD-1/PD-L1 inhibitors monotherapy verse (vs) standard of care (SoC) or placebo, PD-1/PD-L1 inhibitors combined with SoC vs SoC in adult patients with solid tumors were eligible for analysis. The approval of cemiplimab in September 2018 was based on two single-arm trials^17^, and no randomized trials had been reported, so it was not included in present study.

Potentially relevant studies were identified from previous meta-analyses^18^-^20^, and a search of PubMed and Web of Knowledge (through November 30^th,^ 2018), with no limitations on publication year or language restrictions. Abstracts from the major conference proceedings of the American Society of Clinical Oncology, the European Society for Medical Oncology, and the World Conference for Lung Cancer were also searched for unpublished studies. We used search terms “carcinoma”, “cancer”, “trial”, “checkpoint inhibitors”, “PD-1”, “PD-L1” combined with PD-1/PD-L1 inhibitors' names (nivolumab, pembrolizumab, atezolizumab, durvalumab and avelumab). The search strategies were detailed in the supplementary materials. We combined the search results in a bibliographic management tool (EndNote), and eliminated duplicates. After preliminary screening of titles and abstracts, three independent authors (Q-YH, Y-ZZ and Z-DG) assessed the full text for final selection. We also reviewed the references of articles included in the final inclusion.

### Data extraction and quality assessment

Data extraction was carried out using a predefined electronic database. Both OS and PFS were chosen as the outcomes for efficacy, and the HRs and their 95% confidence interval (95%CI) were our preferred outcome measure, because HRs provide time-to-event information and account for censoring. Grade 3-5 treatment-related adverse events (TrAEs) were chosen as the outcomes for safety, and number of patients in safety analysis and grade 3-5 TrAEs of each treatment arm were recorded. Multiple source reports derived from one trial were all included in selection process, and the most updated and complete data were extracted for the analysis. Besides, we also extracted other clinicopathological characteristics of each study, including first author, year of publication or presentation, study name, cancer type, trial phase, line of therapy, treatment regimen, sample size and median of follow-up.

We employed the Jadad scale to quantitatively evaluate the quality of studies. Study selection, data extraction and quality assessment were performed by two of three authors (Q-YH, Y-ZZ and Z-DG). Any discrepancies were resolved by discussion involving all three to achieve consensus.

### Data synthesis and statistical analysis

We estimated summary HRs for time-to-event outcomes, and odds ratios (ORs) for dichotomous outcomes using traditional pairwise and network meta-analysis. Pooled HRs of multiple doses of one drug in one trial were combined by meta-analysis. As the included trials are likely different in many aspects, heterogeneity is generally expected in a meta-analysis. Therefore, the study effect sizes were synthesized using random-effects models to account for heterogeneity.

Bayesian network meta-analysis was performed with the “rjags” package in R (version 3.5.1), which used Markov Chain Monte Carlo (MCMC) algorithm with three chain to generate posterior distribution of model parameters. Each chain had a run of 200,000 updates after a 50,000-run burn-in period with thinning rate 2 for reducing sample autocorrelations. We performed pairwise meta-analysis to synthesize studies comparing the same pair of treatments with Stata 12 (StataCorp, College Station, TX, USA). Excellent consistency, represented by the agreement between indirect and direct comparisons, is the key to robust results. Consistency was evaluated by comparing the pooled HRs from the network meta-analysis with corresponding HRs from pairwise meta-analysis. *P* values (less than 0.05) and 95% CIs/CrIs were used to assess statistical significance. All statistical analyses were two-sided.

## Results

### Study selection and characteristics

**Figure 1:**
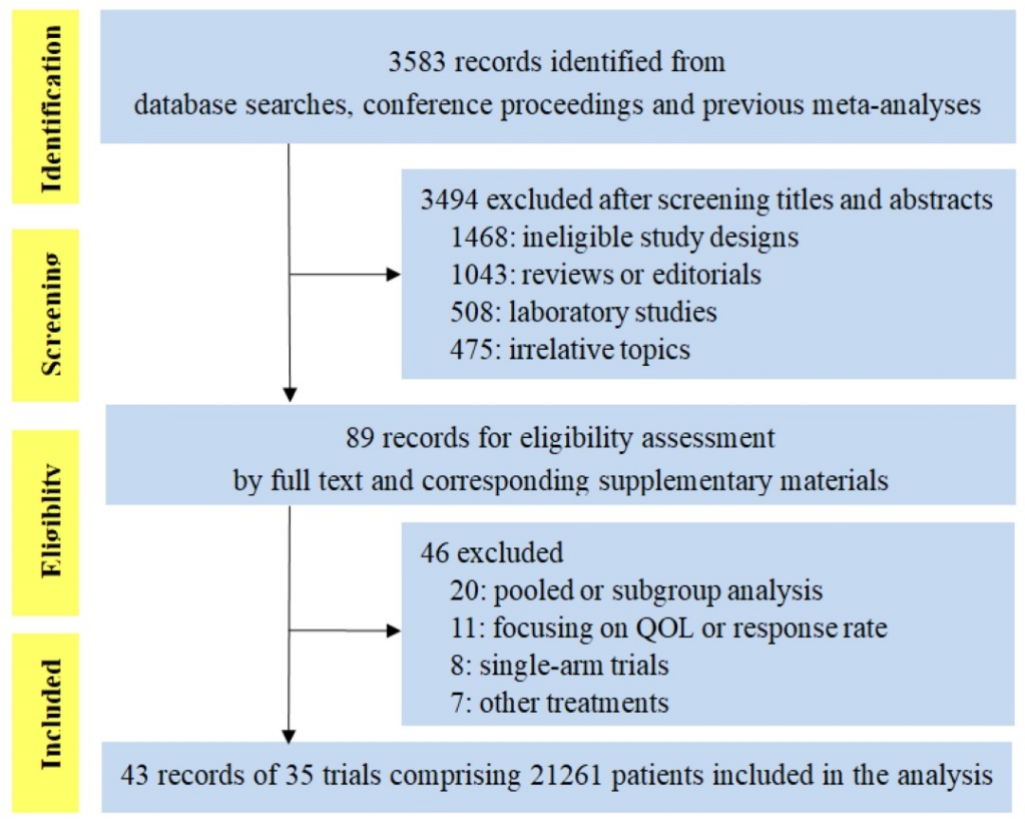
PRISMA flow chart displaying the search and selection process

We identified 3583 citations for review of title and abstract by the search. As shown in Figure 1, the screening process totally included 43 publications reporting 35 trials of 21261 patients, evaluating five PD-1/PD-L1 inhibitors (23 trials of PD-1 inhibitors: 11 of nivolumab and 12 of pembrolizumab; 12 trials of PD-L1 inhibitors: 7 of atezolizumab, 2 of durvalumab and 3 of avelumab). Eight trials were conducted in melanoma, 16 in lung cancer (8 in NSCLC, 5 in non-squamous cell NSCLC, 2 in squamous cell NSCLC and 1 in small-cell lung cancer), 3 in HNSCC, 4 in urinary system cancer, 3 in gastric or gastro-esophageal junction cancer and 1 in triple-negative breast cancer. There were 4 phase 2 trials, 29 phase 3 trials, and 1 phase 2/3 trial. Fourteen trials were done in first-line setting, 17 in second-or subsequent-line setting, 1 in first-or second-line setting and 3 in adjuvant setting. The median score was 3 (range 2-5), with 31 (88.6%) reports receiving high-quality score (Jadad score of 3-5), and the remaining four reports with low-quality score (Jadad score of 1-2) were all meeting abstracts without detailed reports at this moment (Supplementary Table S1).

**Figure 2:**
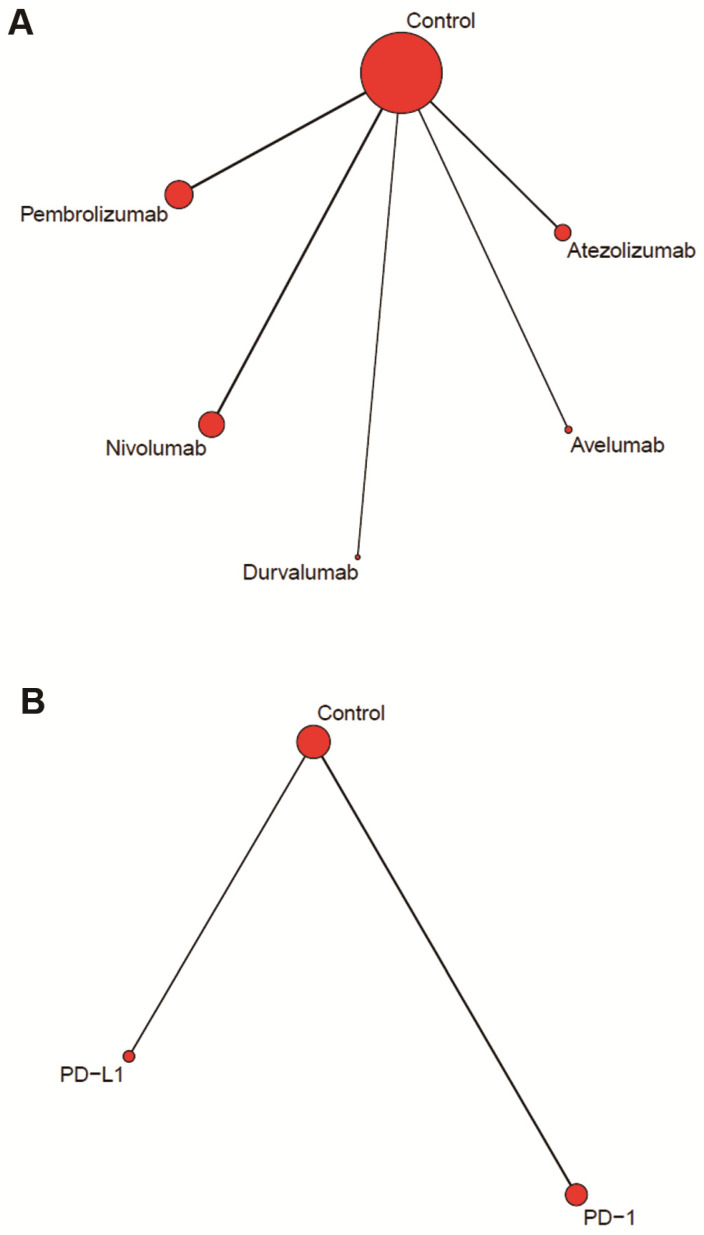
Network plots of comparisons for drug (A) and category (B) based network meta-analyses.

Figure 2A showed the network of drug-based comparison, which was designed for multiple comparison of the five inhibitors. Figure 2B showed the network of category-based comparison, which established the comparison of PD-1 inhibitors (nivolumab and pembrolizumab) and PD-L1 inhibitors (atezolizumab, durvalumab and avelumab).

### Drug-based network meta-analysis

**Figure 3:**
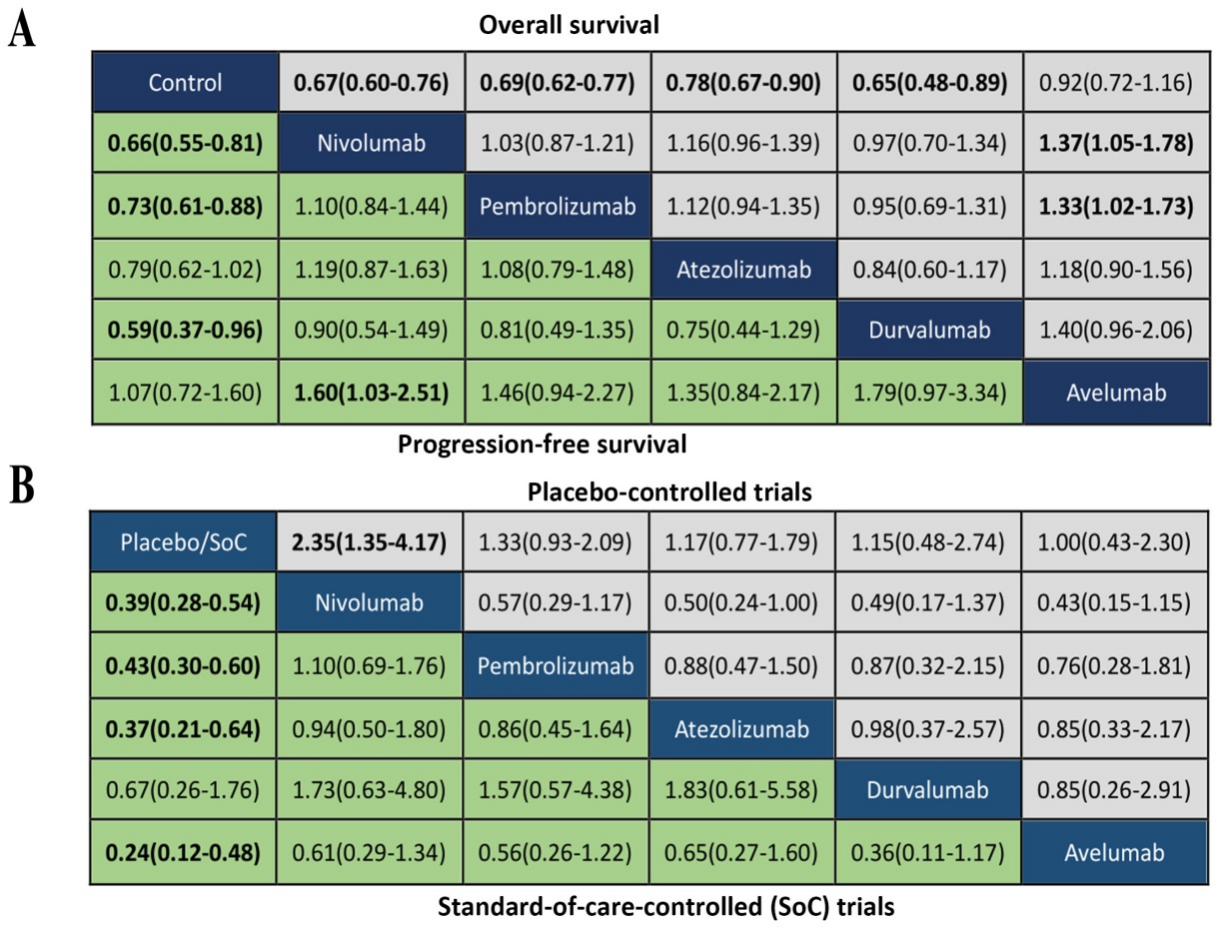
(A) Head-to-head comparisons for overall survival (upper triangle) and progression-free survival (lower triangle) according to the drug-based network meta-analysis. (B) Head-to-head comparisons for ≥Grade 3 treatment-related adverse events according to the drug-based network meta-analysis of placebo-controlled (upper triangle) or standard-of-care-controlled (SoC) trials (lower triangle). Data are hazard ratios (HRs) with their 95% credible interval (95% CrI) in the column-defining treatment compared with the row-defining treatment. HRs higher than 1 favor the column-defining treatment. Significant results are in **bold**.

**Figure 4:**
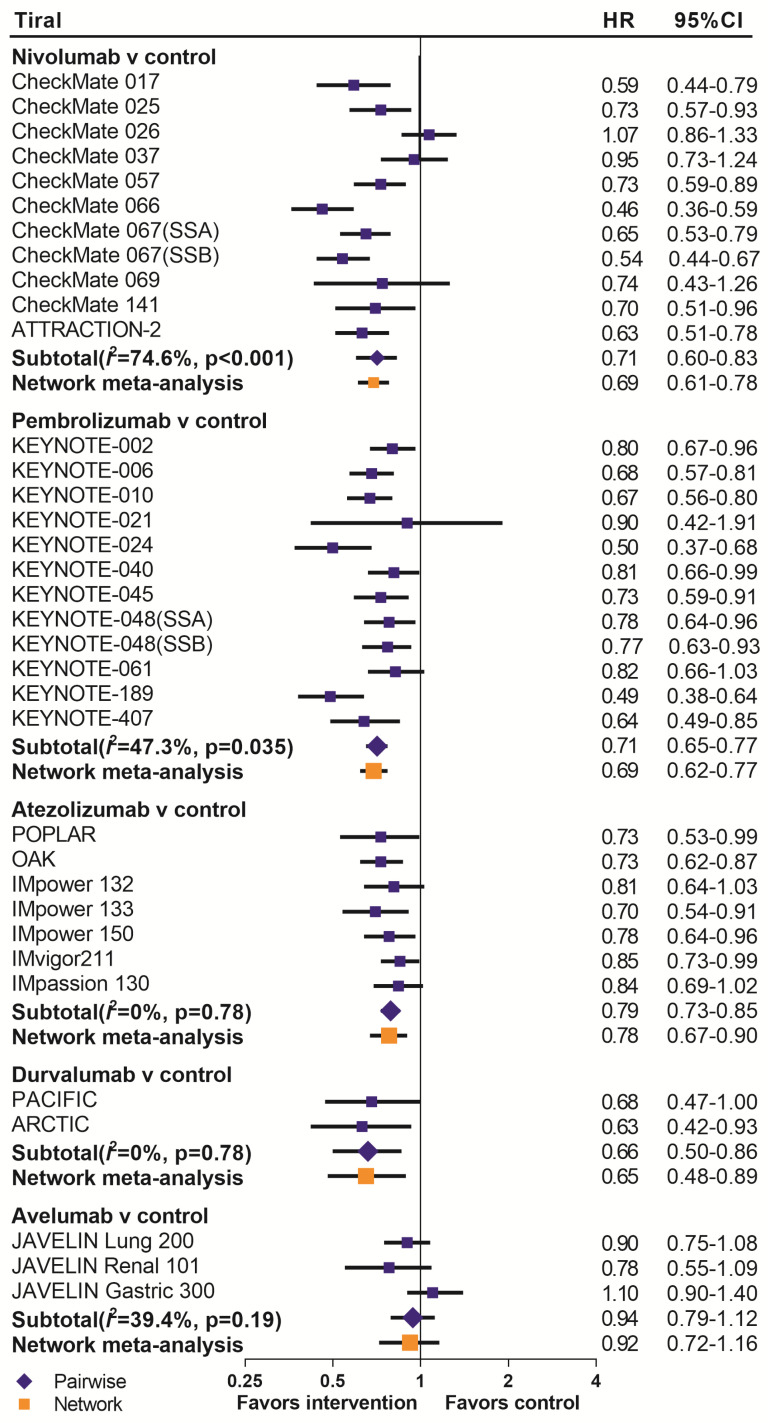
Pooled hazard ratios for overall survival by traditional pairwise meta-analysis (direct comparison) and network meta-analysis (indirect comparison) in the drug-based analysis. HR=hazard ratio; 95%CI=confidence interval for traditional meta-analysis and 95% credible interval for Bayesian network meta-analysis; SSA=sub study A; SSB=sub study B.

We summarized the results of the random-effects Bayesian network meta-analysis for OS and PFS of five inhibitors in Figure 3A. Compared with control group, nivolumab (OS: HR 0.67, 95%CrI 0.60-0.76; PFS: HR 0.66, 95%CrI 0.55-0.81), pembrolizumab (OS: HR 0.69, 95%CrI 0.62-0.77; PFS: HR 0.73, 95%CrI 0.61-0.88) and durvalumab (OS: HR 0.65, 95%CrI 0.48-0.89; PFS: HR 0.59, 95%CrI 0.37-0.97) significantly improved both OS and PFS. Atezolizumab significantly prolonged OS (HR 0.78, 95%CrI 0.67-0.90), but the PFS improvement was not statistically significant (HR 0.79, 95%CrI 0.61-1.02). The comparisons of nivolumab, pembrolizumab, atezolizumab and durvalumab indicated non-significant differences in OS and PFS between any two drugs. Avelumab provided benefits for neither OS (HR 0.92, 95%CrI 0.72-1.16), nor PFS (HR 1.07, 95%CrI 0.72-1.62), compared with control group. Moreover, the OS of avelumab was significantly inferior to nivolumab (HR 1.37, 95%CrI 1.05-1.78) and pembrolizumab (HR 1.33, 95%CrI 1.02-1.73), and the PFS of avelumab was significantly inferior to nivolumab (HR 1.60, 95%CrI 1.03-2.51).The comparison of results from traditional pairwise meta-analysis and network meta-analysis showed outstanding consistency in both significance and tendency (Figure 4).

We performed subgroup analyses according to the line of therapy (Supplementary Figure S1), type of control treatment (Supplementary Figure S2) and cancer type (Supplementary Figure S3). Significant differences in OS and PFS were not noted between any two drugs of nivolumab, pembrolizumab, atezolizumab and durvalumab, except that atezolizumab had significantly unfavorable PFS than nivolumab (HR 1.53, 95%CrI 1.03-2.28) in the placebo-controlled subgroup. Poorer OS and PFS than nivolumab and pembrolizumab were also noted with avelumab, although statistical significance was not reached in some subgroups. It was not applicable to pool the risk of death or progression for some subgroups, as only one trial was available in these subgroups, such as urinary system cancer subgroup and HNSCC subgroups.

The analysis of grade 3-5 TrAEs was conducted in according to the type of control group. As showed in Figure 3B, nivolumab treatment was more likely to cause grade 3-5 TrAEs than placebo (OR 2.35, 95%CrI 1.35-4.17), while other inhibitors were not significantly associated with grade 3-5 TrAEs compared with placebo. Comparisons of the five inhibitors did not reveal any significant differences in grade 3-5 TrAEs between any two inhibitors. Compared with SoC treatment, nivolumab (OR 0.39, 95%CrI 0.28-0.54), pembrolizumab(OR 0.43, 95%CrI 0.30-0.60), atezolizumab(OR 0.37, 95%CrI 0.21-0.64) and avelumab (OR 0.24, 95%CrI 0.12-0.48) were significantly associated with reduction of grade 3-5 TrAEs, expcept durvalumab (OR 0.67, 95%CrI 0.26-1.76). There were not significant differences of grade 3-5 TrAEs between any two inhibitors.

CheckMate 067, CheckMate 069, KEYNOTE-006, and CheckMate 238 trials used ipilimumab, an antibody against CTLA4, as the control regimen. Sensitivity analysis by excluding these trials derived consistent results with the original analysis in terms of survival and toxicity (Supplementary Figure S4).

### Category-based network meta-analysis

**Figure 5:**
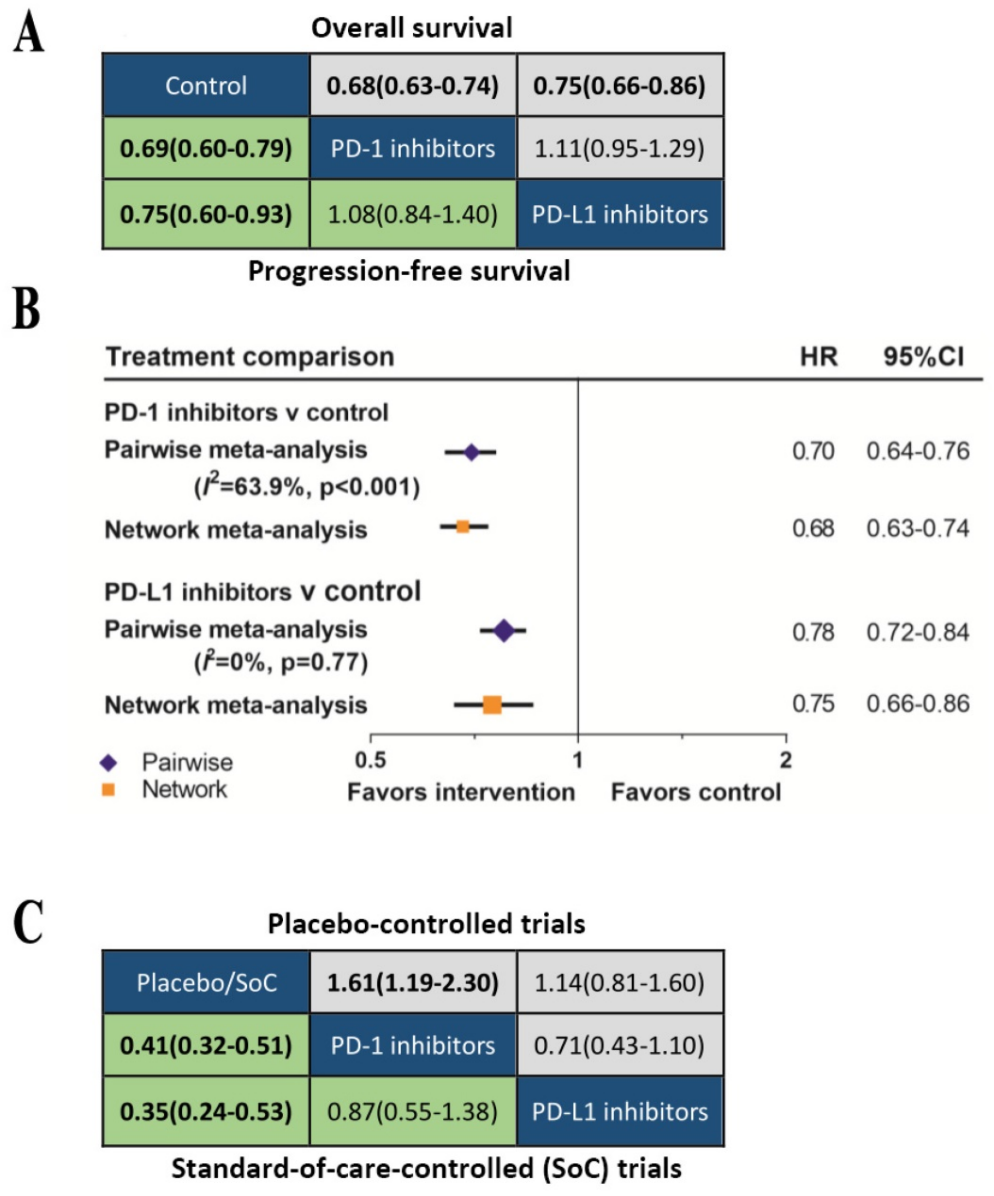
(A) Head-to-head comparisons for overall survival (upper triangle) and progression-free survival (lower triangle) according to the category-based network meta-analysis. (B) Pooled hazard ratios for overall survival by traditional pairwise meta-analysis (direct comparison) and network meta-analysis (indirect comparison) in the category-based analysis. (C) Head-to-head comparisons for ≥Grade 3 treatment-related adverse events according to the category-based network meta-analysis of placebo-controlled (upper triangle) or standard-of-care-controlled (SoC) trials (lower triangle).

We further classified the drugs as PD-1 inhibitors or PD-L1 inhibitors according to the treatment target. The network meta-analysis (Figure 5A) showed that both PD-1 inhibitors (OS: HR 0.69, 95%CrI 0.64-0.75; PFS: HR 0.69, 95%CrI 0.60-0.79) and PD-L1 inhibitors (OS: HR 0.79, 95%CrI 0.70-0.88; PFS: HR 0.81, 95%CrI 0.67-0.99) provided OS and DFS advantage over control group. The Bayesian-based comparison between PD-1 inhibitors and PD-L1 inhibitors did not imply significant difference in OS (HR 1.14, 95%CrI 0.99-1.31) and PFS (HR 1.18, 95%CrI 0.93-1.50). Comparing results from pairwise meta-analysis and network meta-analysis did not suggest inconsistency (figure 5B). Subgroup analyses according to the line of therapy (Supplementary Figure S5), type of control treatment (Supplementary Figure S6) and cancer type (Supplementary Figure S7) were also performed. We did not find significant difference in OS or PFS between PD-1 inhibitors and PD-L1 inhibitors in all subgroups. Previous analysis indicated that the efficacy of avelumab was inferior to nivolumab and pembrolizumab, therefore, we excluded avelumab and re-analyzed the data. Re-analyasis excluding trials of avelumab did not show any significant differences in OS (HR 1.11, 95%CrI 0.95-1.29) or PFS (HR 1.08, 95%CrI 0.84-1.40) between PD-1 inhibitors and PD-L1 inhibitors.

The comparisons of safety for these two types of inhibitors were summarized in Figure 5B. Among the placebo-controlled trials, the risk of grade 3-5 TrAEs between PD-1 inhibitors and PD-L1 inhibitors was comparable (OR 0.71, 95%CrI 0.43-1.10). Compared with SoC, both PD-1 inhibitors (OR 0.41, 95%CrI 0.32-0.51) and PD-L1 inhibitors (OR 0.35, 95%CrI 0.24-0.53) were safer, and the safety of PD-1 inhibitors and PD-L1 inhibitors were equivalent (OR 0.87, 95%CrI 0.55-1.38).

Sensitivity analysis excluding CheckMate 067, CheckMate 069, KEYNOTE-006, and CheckMate 238 trials showed that PD-1 inhibitors and PD-L1 inhibitors were equivalent in survival and of grade 3-5 TrAEs (Supplementary Figure S8).

## Discussion

The clinical use of anti-PD-1/PD-L1 immunotherapy is rapidly expanding, and more drugs are striving to enter the game^57^, however, little comparative evidence of the inhibitors is currently available. Our Bayesian network meta-analysis compares five FDA-approved PD-1/PD-L1 inhibitors (cemiplimab was excluded for unavailable randomized trials), and analyzes both survival and toxicity of the inhibitors. We found that all PD-1/PD-L1 inhibitors, except avelumab, were more efficacious than control treatment in patients with solid tumors. Significant inferior survival was observed among patients treated with avelumab than those with nivolumab and pembrolizumab, while the other four inhibitors treatment yielded equivalent survival. These findings were further strengthened by their consistency across all analyzed subgroups, as the large sample size allowed thorough subgroup analyses. Analysis of toxicity demonstrated no significant differences in the risk of grade 3-5 TrAEs between any two inhibitors. It was noteworthy that all five inhibitors were generally associated with lower frequency of TrAEs when compared with SoC, making them more favorable options in the clinic.

Avelumab was approved by FDA for metastatic MCC and urothelial carcinoma based on its excellent performance in two single-arm trials. Among 88 patients with treated MCC who were enrolled for avelumab treatment, 32% experienced complete or partial remission of their tumors, and responses were ongoing in 82% of responding patients at the time of analysis 58. FDA granted avelumab an accelerated approval and orphan drug designation to fill an unmet medical need for MCC. The approval for urothelial carcinoma was based on data of 242 patients, of whom the overall response rate (ORR) was 13.3% among those who were followed for at least 13 weeks, and 16.1% among who were followed for at least 6 months 59. Nevertheless, avelumab treatment failed to reach the study endpoint of OS or PFS improvement among patients with NSCLC 37 and gastric or gastro-esophageal junction cancer 55 in the subsequent-line setting. Considering these results, our findings are reasonable that the survival with avelumab treatment was not superior to that with chemotherapy, and inferior to that with nivolumab and pembrolizumab treatment. These results indicate that PD-1/PD-L1 inhibitors are not equally efficacious, and the efficacy of an inhibitor may vary with cancer type. These inhibitors should not be simply regarded as one entity, although they principally act through blocking PD-1/PD-L1 pathway. More attention should be paid to this in clinical practice and design of clinical studies.

To the best of our knowledge, the present study has been the most comprehensive and updated meta-analysis so far, and is the first one to directly reveal the disparity in survival of PD-1/PD-L1 inhibitors. Two indirect comparisons by Passiglia *et al* 60 and Créquit *et al* 61 both indicate the equivalent OS and PFS of nivolumab, pembrolizumab and atezolizumab. The network meta-analysis by You *et al* 62 show no significant difference in survival benefits between PD-1 inhibitors and PD-L1 inhibitors. Our results are in accordance with the previous analyses, however, they only include three inhibitors, and miss durvalumab and avelumab. These analyses also have quite poor generalizability because their study design are only limited to NSCLC in the subsequent-line setting. Another strength of our work is that the analysis is performed through Bayesian approach. Frequentist approach, which is used in the studies of Passiglia *et al* 60 and You *et al* 62, relies on the traditional statistical methods. By contrast, Bayesian approach is based on a solid mathematical foundation and a model-based framework, and results are calculated with MCMC simulations, allowing model reproduction several times until convergence. It has been widely acknowledged that Bayesian methods are more flexible, and the results are more clinically interpretable, so they are employed in more network meta-analyses^63^.

In addition, this work is also strengthen by the quality and quantity of studies identified and used in the pooled analysis. Data are obtained from 35 published and unpublished randomized controlled trials, of which 88.6% achieve high-quality Jadad scores. OS and PFS are used to measure the efficacy outcomes, and grade 3-5 TrAEs to the safety outcomes. These endpoints are well-defined in clinical trials. The large number of included trials without a limitation of cancer type is a crucial point. Recently-approved drugs, such as durvalumab and avelumb, have a small number of randomized trials at this moment. Our high quality and large quantity of data lead to good statistical power, and make the results reliable. Finally, all pooled HRs and their CrIs of network meta-analysis are close to the corresponding results of pairwise meta-analysis, supporting that there is no significant inconsistency between direct and indirect comparisons.

Our work has some limitations. First of all, the present analysis included all solid tumors with different control treatment regimens, and this would introduce heterogeneity to the results. To address this issue, we performed detailed subgroup analyses, and generate same results. Additionally, two trials of PD-1 inhibitors as adjuvant treatment, CheckMate 238 4 and KEYNOTE-054 3, only reported recurrence-free survival results, which are used as surrogate endpoint of PFS in our analysis. The OS data are immature, thus unavailable for the synthesis of. Lastly, this meta-analysis was conducted with summary statistics rather than individual participant data, so it could not be adjusted for some potential covariates.

In summary, our large-scale network meta-analysis based on Bayesian approach revealed the disparity in the efficacy of PD-1/PD-L1 inhibitors. The survival of treatment with nivolumab, pembrolizumab, atezolizumab and durvalumab were equivalent in solid tumors, while avelumab was associated with unfarvorable survival. PD-1/PD-L1 inhibitors were comparable in the risk of TrAEs, and safer than conventional therapies. These results could assist in the decision-making of clinicians and their patients, and provide more information for the future clinical studies.

## Supplementary Material

Supplementary information, figures and table.

## Figures and Tables

**Table 1:** Clinicopathologic characteristics of 35 randomized controlled trials included for analysis

First author, year		Trial name	Phase	Line of therapy	Treatment	Drug target	Control type	PD-L1 expression	Sample size	Median follow-up(mo)	Jadad score
Melanoma											
	Ribas,2015^21^/Hamid,2017^22^	KEYNOTE-002	2	2nd	Pembrolizumab v ICC(paclitaxel+carboplatin, paclitaxel, carboplatin, dacarbazine or temozolomide).	PD-1	SoC	*NA*	540	28	3
	Weber,2015^23^/Larkin2018^24^	CheckMate 037	3	2nd/ subsequent	Nivolumab v ICC(dacarbazine or paclitaxel+carboplatin)	PD-1	SoC	*NA*	405	24	3
	Robert,2015^25^/Ascierto,2018^26^	CheckMate 066	3	1st	Nivolumab v dacarbazine	PD-1	SoC	*NA*	418	38.4	5
	Larkin,2015^27^/Wolchok,2017^28^/Hodi,2018^29^	CheckMate 067 (SSA)	3	1st	Nivolumab v ipilimumab	PD-1	SoC	*NA*	631	48^#^	5
		CheckMate 067 (SSB)	3	1st	Nivolumab+ipilimumab v ipilimumab	PD-1	Placebo	*NA*	629	48^#^	5
	Postow,2015^30^/Hodi,2016^31^	CheckMate 069	2	1st	Nivolumab+ipilimumab v ipilimumab	PD-1	Placebo	*NA*	142	24.5	5
	Robert,2015^32^/Schachter, 2017^33^/Carlino,2018^34^	KEYNOTE-006	3	1st/2nd	Pembrolizumab v ipilimumab	PD-1	SoC	*NA*	834	22.9	3
	Weber,2017^4^	CheckMate 238	3	Adjuvant	Nivolumab v ipilimumab	PD-1	SoC	*NA*	906	19.5	5
	Eggermont,2018^3^	KEYNOTE-054	3	Adjuvant	Pembrolizumab v placebo	PD-1	Placebo	*NA*	1019	15	3
Lung cancer											
Non-small-cell lung cancer											
	Herbst,2016^10^	KEYNOTE-010	2/3	2nd/ subsequent	Pembrolizumab v docetaxel	PD-1	SoC	TPS≥1%(22C3 pharmDx *assay)*	1034	13.1	3
	Reck,2016^14^	KEYNOTE-024	3	1st	Pembrolizumab v ICC(platinum doublet)	PD-1	SoC	TPS≥50% (22C3 pharmDx assay)	305	11.2	3
	Fehrenbacher,2016^35^	POPLAR	2	2nd/3rd	Atezolizumab v docetaxel	PD-L1	SoC	*NA*	287	14.8	3
	Rittmeyer,2017^13^	OAK	3	2nd/3rd	Atezolizumab v docetaxel	PD-L1	SoC	*NA*	850	21	3
	Antonia,2017^36^	PACIFIC	3	Adjuvant	Durvalumab v placebo(after chemoradiotherapy)	PD-L1	Placebo	*NA*	709	25.2	5
	Carbone,2018^15^	CheckMate 026	3	1st	Nivolumab v ICC(platinum doublet)	PD-1	SoC	≥1% (28-8 pharmDx assay)	541	13.5	3
	Barlesi,2018^37^	JAVELIN Lung 200	3	2nd	Avelumab v docetaxel	PD-L1	SoC	*NA*	792	18.3	3
	Kowalski,2018^38^	ARCTIC	3	3rd/ subsequent	Durvalumab v erlotinib, gemcitabine or vinorelbine	PD-L1	SoC	TC≥25% (Ventana SP263 assay)	126	*NR*	2
Non-squamous cell non-small-cell lung cancer											
	Borghaei,2015^39^	CheckMate 057	3	2nd/3rd	Nivomulab v docetaxel	PD-1	SoC	*NA*	582	13.2^#^	3
	Langer,2016^40^	KEYNOTE-021	2	1st	Pembrolizumab+carboplatin+pemetrexed v carboplatin+pemetrexed	PD-1	Placebo	*NA*	123	10.6	3
	Grandhi,2018^41^	KEYNOTE-189	3	1st	Pembrolizumab+chemotherapy(pemetrexed+platinum) v chemotherapy	PD-1	Placebo	*NA*	616	10.5	5
	Papadimitrakopoulou,2018^42^	IMpower 132	3	1st	Atezolizumab+chemotherapy (cisplatin or carboplatin+pemetrexed) v chemotherapy	PD-L1	Placebo	*NA*	578	14.8	2
	Socinski,2018^43^	IMpower 150	3	1st	Atezolizumab+BCP(bevacizumab+ carboplatin+paclitaxel) v BCP	PD-L1	Placebo	*NA*	692	15.5	3
Squamous cell non-small-cell lung cancer											
	Brahmer,2015^12^	CheckMate 017	3	2nd	Nivomulab v docetaxel	PD-1	SoC	*NA*	272	11^#^	3
	Paz‑Ares,2018^44^	KEYNOTE-407	3	1st	Pembrolizumab+chemotherapy(carboplatin+paclitaxel or nab-paclitaxel) v chemotherapy	PD-1	Placebo	*NA*	559	7.8	5
Small cell lung cancer											
	Horn,2018^45^	IMpower 133	3	1st	Atezolizumab+carboplatin+etoposide v carboplatin+etoposide	PD-L1	Placebo	*NA*	403	13.9	5
Head and neck squamous cell carcinoma											
	Ferris,2016^46^	CheckMate 141	3	2nd/ subsequent	Nivolumab v methotrexate, docetaxel, or cetuximab	PD-1	SoC	*NA*	361	5.1	3
	Cohen,2017^47^	KEYNOTE-040	3	2nd	Pembrolizumab v methotrexate, docetaxel or cetuximab	PD-1	SoC	*NA*	495	7.3	3
	Burtness,2018^48^	KEYNOTE-048 (SSA)	3	1st	Pembrolizumab v EXTREME (cetuximab+platinum+5-FU)	PD-1	SoC	CPS≥1%	512	17^#^	2
		KEYNOTE-048 (SSB)	3	1st	Pembrolizumab+platinum+5-FU v EXTREME	PD-1	Placebo	*NA*	559	17^#^	2
Urinary system cancer											
Urothelial cancer											
	Bellmunt,2017^49^	KEYNOTE-045	3	2nd/ subsequent	Pembrolizumab v ICC(paclitaxel, docetaxel, or vinflunine)	PD-1	SoC	*NA*	542	14.1	3
	Powles,2018^50^	IMvigor211	3	2nd/ subsequent	Atezolizumab v ICC(vinflunine, paclitaxel, or docetaxel)	PD-L1	SoC	*NA*	931	17.3	3
Renal cell carcinoma											
	Motzer,2015^51^	CheckMate 025	3	2nd/subsequent	Nivolumab v everolimus	PD-1	SoC	*NA*	821	14^#^	3
	Motzer,2018^52^	JAVELIN Renal 101	3	1st	Avelumab+axitinib v sunitinib	PD-L1	Placebo	*NA*	886	*NR*	2
Gastric or gastro-esophageal junction cancer											
	Kang,2017^53^	ATTRACTION-2	3	3rd/ subsequent	Nivomulab v placebo	PD-1	Placebo	*NA*	493	8.87	5
	Shitara,2018^54^	KEYNOTE-061	3	2nd	Pembrolizumab versus paclitaxel	PD-1	SoC	PD-L1 CPS≥1%	395	7.9	3
	Bang,2018^55^	JAVELIN Gastric 300	3	3rd	Avelumab versus ICC(paclitaxel or irinotecan)	PD-L1	SoC	*NA*	371	10.6	3
Triple-negative breast cancer											
	Schmid,2018^56^	IMpassion 130	3	1st	Atezolizumab+nab-paclitaxel v nab-paclitaxel	PD-L1	Placebo	*NA*	902	12.9	5

Abbreviations: *NA*=not applicable; *NR*=not reported; PD-L1=programmed cell death ligand 1; mo=month; PD-1=programmed cell death 1; SSA=sub study A, SSB=sub study B

## References

[1] Sanmamed MF, Chen L (2018). A Paradigm Shift in Cancer Immunotherapy: From Enhancement to Normalization. Cell.

[2] Sharma P, Hu-Lieskovan S, Wargo JA, Ribas A (2017). Primary, Adaptive, and Acquired Resistance to Cancer Immunotherapy. Cell.

[3] Eggermont AMM, Blank CU, Mandala M, Long GV, Atkinson V, Dalle S (2018). Adjuvant Pembrolizumab versus Placebo in Resected Stage III Melanoma. New England Journal of Medicine.

[4] Weber J, Mandala M, Del Vecchio M, Gogas HJ, Arance AM, Cowey CL (2017). Adjuvant Nivolumab versus Ipilimumab in Resected Stage III or IV Melanoma. New England Journal of Medicine.

[5] Pardoll DM (2012). The blockade of immune checkpoints in cancer immunotherapy. Nature reviews Cancer.

[6] Butte MJ, Keir ME, Phamduy TB, Sharpe AH, Freeman GJ (2007). Programmed death-1 ligand 1 interacts specifically with the B7-1 costimulatory molecule to inhibit T cell responses. Immunity.

[7] Azuma T, Yao S, Zhu G, Flies AS, Flies SJ, Chen L (2008). B7-H1 is a ubiquitous antiapoptotic receptor on cancer cells. Blood.

[8] Clark CA, Gupta HB, Sareddy G, Pandeswara S, Lao S, Yuan B (2016). Tumor-Intrinsic PD-L1 Signals Regulate Cell Growth, Pathogenesis, and Autophagy in Ovarian Cancer and Melanoma. Cancer Res.

[9] Boyerinas B, Jochems C, Fantini M, Heery CR, Gulley JL, Tsang KY (2015). Antibody-Dependent Cellular Cytotoxicity Activity of a Novel Anti-PD-L1 Antibody Avelumab (MSB0010718C) on Human Tumor Cells. Cancer Immunol Res.

[10] Herbst RS, Baas P, Kim DW, Felip E, Perez-Gracia JL, Han JY (2016). Pembrolizumab versus docetaxel for previously treated, PD-L1-positive, advanced non-small-cell lung cancer (KEYNOTE-010): a randomised controlled trial. Lancet.

[11] Garon EB, Rizvi NA, Hui R, Leighl N, Balmanoukian AS, Eder JP (2015). Pembrolizumab for the treatment of non-small-cell lung cancer. The New England journal of medicine.

[12] Brahmer J, Reckamp KL, Baas P, Crino L, Eberhardt WE, Poddubskaya E (2015). Nivolumab versus Docetaxel in Advanced Squamous-Cell Non-Small-Cell Lung Cancer. The New England journal of medicine.

[13] Rittmeyer A, Barlesi F, Waterkamp D, Park K, Ciardiello F, von Pawel J (2017). Atezolizumab versus docetaxel in patients with previously treated non-small-cell lung cancer (OAK): a phase 3, open-label, multicentre randomised controlled trial. Lancet.

[14] Reck M, Rodriguez-Abreu D, Robinson AG, Hui R, Csoszi T, Fulop A (2016). Pembrolizumab versus Chemotherapy for PD-L1-Positive Non-Small-Cell Lung Cancer. The New England journal of medicine.

[15] Carbone DP, Reck M, Paz-Ares L, Creelan B, Horn L, Steins M (2017). First-Line Nivolumab in Stage IV or Recurrent Non-Small-Cell Lung Cancer. The New England journal of medicine.

[16] Liao W-C, Chien K-L, Lin Y-L, Wu M-S, Lin J-T, Wang H-P (2013). Adjuvant treatments for resected pancreatic adenocarcinoma: a systematic review and network meta-analysis. The Lancet Oncology.

[17] Migden MR, Rischin D, Schmults CD, Guminski A, Hauschild A, Lewis KD (2018). PD-1 Blockade with Cemiplimab in Advanced Cutaneous Squamous-Cell Carcinoma. The New England journal of medicine.

[18] Huang Q, Zhang H, Hai J, Socinski MA, Lim E, Chen H (2018). Impact of PD-L1 expression, driver mutations and clinical characteristics on survival after anti-PD-1/PD-L1 immunotherapy versus chemotherapy in non-small-cell lung cancer: A meta-analysis of randomized trials. Oncoimmunology.

[19] Xu C, Chen Y-P, Du X-J, Liu J-Q, Huang C-L, Chen L (2018). Comparative safety of immune checkpoint inhibitors in cancer: systematic review and network meta-analysis. Bmj.

[20] Baxi S, Yang A, Gennarelli RL, Khan N, Wang Z, Boyce L (2018). Immune-related adverse events for anti-PD-1 and anti-PD-L1 drugs: systematic review and meta-analysis. Bmj.

[21] Liu B, Song Y, Liu D (2017). Recent development in clinical applications of PD-1 and PD-L1 antibodies for cancer immunotherapy. Journal of hematology & oncology.

[22] Kaufman HL, Russell J, Hamid O, Bhatia S, Terheyden P, D'Angelo SP (2016). Avelumab in patients with chemotherapy-refractory metastatic Merkel cell carcinoma: a multicentre, single-group, open-label, phase 2 trial. The Lancet Oncology.

[23] Three Drugs Approved for Urothelial Carcinoma by FDA Cancer discovery. 2017; 7: 659-60.

[24] Barlesi F, Vansteenkiste J, Spigel D, Ishii H, Garassino M, de Marinis F (2018). Avelumab versus docetaxel in patients with platinum-treated advanced non-small-cell lung cancer (JAVELIN Lung 200): an open-label, randomised, phase 3 study. The Lancet Oncology.

[25] Bang YJ, Ruiz EY, Van Cutsem E, Lee KW, Wyrwicz L, Schenker M (2018). Phase III, randomised trial of avelumab versus physician's choice of chemotherapy as third-line treatment of patients with advanced gastric or gastro-oesophageal junction cancer: primary analysis of JAVELIN Gastric 300. Annals of oncology: official journal of the European Society for Medical Oncology.

[26] Passiglia F, Galvano A, Rizzo S, Incorvaia L, Listi A, Bazan V (2018). Looking for the best immune-checkpoint inhibitor in pre-treated NSCLC patients: An indirect comparison between nivolumab, pembrolizumab and atezolizumab. Int J Cancer.

[27] Crequit P, Chaimani A, Yavchitz A, Attiche N, Cadranel J, Trinquart L (2017). Comparative efficacy and safety of second-line treatments for advanced non-small cell lung cancer with wild-type or unknown status for epidermal growth factor receptor: a systematic review and network meta-analysis. BMC Med.

[28] You W, Liu M, Miao J-D, Liao Y-Q, Song Y-B, Cai D-K (2018). A Network Meta-analysis Comparing the Efficacy and Safety of Anti-PD-1 with Anti-PD-L1 in Non-small Cell Lung Cancer. Journal of Cancer.

[29] Hong H, Carlin BP, Shamliyan TA, Wyman JF, Ramakrishnan R, Sainfort F (2013). Comparing Bayesian and frequentist approaches for multiple outcome mixed treatment comparisons. Medical decision making: an international journal of the Society for Medical Decision Making.

[30] Ribas A, Puzanov I, Dummer R, Schadendorf D, Hamid O, Robert C (2015). Pembrolizumab versus investigator-choice chemotherapy for ipilimumab-refractory melanoma (KEYNOTE-002): a randomised, controlled, phase 2 trial. The Lancet Oncology.

[31] Hamid O, Puzanov I, Dummer R, Schachter J, Daud A, Schadendorf D (2017). Final analysis of a randomised trial comparing pembrolizumab versus investigator-choice chemotherapy for ipilimumab-refractory advanced melanoma. Eur J Cancer.

[32] Weber JS, D'Angelo SP, Minor D, Hodi FS, Gutzmer R, Neyns B (2015). Nivolumab versus chemotherapy in patients with advanced melanoma who progressed after anti-CTLA-4 treatment (CheckMate 037): a randomised, controlled, open-label, phase 3 trial. The Lancet Oncology.

[33] Larkin J, Minor D, D'Angelo S, Neyns B, Smylie M, Miller WH Jr (2018). Overall Survival in Patients With Advanced Melanoma Who Received Nivolumab Versus Investigator's Choice Chemotherapy in CheckMate 037: A Randomized, Controlled, Open-Label Phase III Trial. Journal of clinical oncology: official journal of the American Society of Clinical Oncology.

[34] Robert C, Long GV, Brady B, Dutriaux C, Maio M, Mortier L (2015). Nivolumab in previously untreated melanoma without BRAF mutation. The New England journal of medicine.

[35] Ascierto PA, Long GV, Robert C, Brady B, Dutriaux C, Di Giacomo AM (2018). Survival Outcomes in Patients With Previously Untreated BRAF Wild-Type Advanced Melanoma Treated With Nivolumab Therapy: Three-Year Follow-up of a Randomized Phase 3 Trial. JAMA Oncol.

[36] Larkin J, Chiarion-Sileni V, Gonzalez R, Grob JJ, Cowey CL, Lao CD (2015). Combined Nivolumab and Ipilimumab or Monotherapy in Untreated Melanoma. The New England journal of medicine.

[37] Wolchok JD, Chiarion-Sileni V, Gonzalez R, Rutkowski P, Grob JJ, Cowey CL (2017). Overall Survival with Combined Nivolumab and Ipilimumab in Advanced Melanoma. The New England journal of medicine.

[38] Hodi FS, Chiarion-Sileni V, Gonzalez R, Grob J-J, Rutkowski P, Cowey CL (2018). Nivolumab plus ipilimumab or nivolumab alone versus ipilimumab alone in advanced melanoma (CheckMate 067): 4-year outcomes of a multicentre, randomised, phase 3 trial. The Lancet Oncology.

[39] Postow MA, Chesney J, Pavlick AC, Robert C, Grossmann K, McDermott D (2015). Nivolumab and ipilimumab versus ipilimumab in untreated melanoma. The New England journal of medicine.

[40] Hodi FS, Chesney J, Pavlick AC, Robert C, Grossmann KF, McDermott DF (2016). Combined nivolumab and ipilimumab versus ipilimumab alone in patients with advanced melanoma: 2-year overall survival outcomes in a multicentre, randomised, controlled, phase 2 trial. The Lancet Oncology.

[41] Robert C, Schachter J, Long GV, Arance A, Grob JJ, Mortier L (2015). Pembrolizumab versus Ipilimumab in Advanced Melanoma. The New England journal of medicine.

[42] Schachter J, Ribas A, Long GV, Arance A, Grob J-J, Mortier L (2017). Pembrolizumab versus ipilimumab for advanced melanoma: final overall survival results of a multicentre, randomised, open-label phase 3 study (KEYNOTE-006). The Lancet.

[43] Carlino MS, Long GV, Schadendorf D, Robert C, Ribas A, Richtig E (2018). Outcomes by line of therapy and programmed death ligand 1 expression in patients with advanced melanoma treated with pembrolizumab or ipilimumab in KEYNOTE-006: A randomised clinical trial. Eur J Cancer.

[44] Fehrenbacher L, Spira A, Ballinger M, Kowanetz M, Vansteenkiste J, Mazieres J (2016). Atezolizumab versus docetaxel for patients with previously treated non-small-cell lung cancer (POPLAR): a multicentre, open-label, phase 2 randomised controlled trial. Lancet.

[45] Antonia SJ, Villegas A, Daniel D, Vicente D, Murakami S, Hui R (2017). Durvalumab after Chemoradiotherapy in Stage III Non-Small-Cell Lung Cancer. The New England journal of medicine.

[46] Kowalski DM, Soo RA, Park K, McCleod M, Geater SL, Powell M (2018). 1378OARCTIC: Durvalumab + tremelimumab and durvalumab monotherapy vs SoC in ≥ 3L advanced NSCLC treatment. Annals of Oncology.

[47] Borghaei H, Paz-Ares L, Horn L, Spigel DR, Steins M, Ready NE (2015). Nivolumab versus Docetaxel in Advanced Nonsquamous Non-Small-Cell Lung Cancer. The New England journal of medicine.

[48] Langer CJ, Gadgeel SM, Borghaei H, Papadimitrakopoulou VA, Patnaik A, Powell SF (2016). Carboplatin and pemetrexed with or without pembrolizumab for advanced, non-squamous non-small-cell lung cancer: a randomised, phase 2 cohort of the open-label KEYNOTE-021 study. The Lancet Oncology.

[49] Gandhi L, Rodriguez-Abreu D, Gadgeel S, Esteban E, Felip E, De Angelis F (2018). Pembrolizumab plus Chemotherapy in Metastatic Non-Small-Cell Lung Cancer. The New England journal of medicine.

[50] Papadimitrakopoulou V, Cobo M, Bordoni R, Dubray-Longeras P, Szalai Z, Ursol G (2018). OA05.07 IMpower132: PFS and Safety Results with 1L Atezolizumab + Carboplatin/Cisplatin + Pemetrexed in Stage IV Non-Squamous NSCLC. Journal of Thoracic Oncology.

[51] Socinski MA, Jotte RM, Cappuzzo F, Orlandi F, Stroyakovskiy D, Nogami N (2018). Atezolizumab for First-Line Treatment of Metastatic Nonsquamous NSCLC. The New England journal of medicine.

[52] Paz-Ares L, Luft A, Vicente D, Tafreshi A, Gümüş M, Mazières J (2018). Pembrolizumab plus Chemotherapy for Squamous Non-Small-Cell Lung Cancer. New England Journal of Medicine.

[53] Horn L, Mansfield AS, Szczesna A, Havel L, Krzakowski M, Hochmair MJ (2018). First-Line Atezolizumab plus Chemotherapy in Extensive-Stage Small-Cell Lung Cancer. The New England journal of medicine.

[54] Ferris RL, Blumenschein G Jr, Fayette J, Guigay J, Colevas AD, Licitra L (2016). Nivolumab for Recurrent Squamous-Cell Carcinoma of the Head and Neck. The New England journal of medicine.

[55] Cohen EEW, Soulières D, Le Tourneau C, Dinis J, Licitra L, Ahn M-J (2019). Pembrolizumab versus methotrexate, docetaxel, or cetuximab for recurrent or metastatic head-and-neck squamous cell carcinoma (KEYNOTE-040): a randomised, open-label, phase 3 study. The Lancet.

[56] Burtness B, Bratland Å, Fuereder T, Hughes BGM, Mesia R, Ngamphaiboon N (2018). LBA8_PRKEYNOTE-048: Phase III study of first-line pembrolizumab (P) for recurrent/metastatic head and neck squamous cell carcinoma (R/M HNSCC). Annals of Oncology.

[57] Bellmunt J, de Wit R, Vaughn DJ, Fradet Y, Lee JL, Fong L (2017). Pembrolizumab as Second-Line Therapy for Advanced Urothelial Carcinoma. The New England journal of medicine.

[58] Powles T, Durán I, van der Heijden MS, Loriot Y, Vogelzang NJ, De Giorgi U (2018). Atezolizumab versus chemotherapy in patients with platinum-treated locally advanced or metastatic urothelial carcinoma (IMvigor211): a multicentre, open-label, phase 3 randomised controlled trial. The Lancet.

[59] Motzer RJ, Escudier B, McDermott DF, George S, Hammers HJ, Srinivas S (2015). Nivolumab versus Everolimus in Advanced Renal-Cell Carcinoma. The New England journal of medicine.

[60] Motzer RJ, Lee JL, Gurney H, Berger R, Schmidinger M, Larkin J (2018). LBA6_PRJAVELIN renal 101: A randomized, phase III study of avelumab + axitinib vs sunitinib as first-line treatment of advanced renal cell carcinoma (aRCC). Annals of Oncology.

[61] Kang Y-K, Boku N, Satoh T, Ryu M-H, Chao Y, Kato K (2017). Nivolumab in patients with advanced gastric or gastro-oesophageal junction cancer refractory to, or intolerant of, at least two previous chemotherapy regimens (ONO-4538-12, ATTRACTION-2): a randomised, double-blind, placebo-controlled, phase 3 trial. The Lancet.

[62] Shitara K, Özgüroğlu M, Bang Y-J, Di Bartolomeo M, Mandalà M, Ryu M-H (2018). Pembrolizumab versus paclitaxel for previously treated, advanced gastric or gastro-oesophageal junction cancer (KEYNOTE-061): a randomised, open-label, controlled, phase 3 trial. The Lancet.

[63] Schmid P, Adams S, Rugo HS, Schneeweiss A, Barrios CH, Iwata H (2018). Atezolizumab and Nab-Paclitaxel in Advanced Triple-Negative Breast Cancer. The New England journal of medicine.

