# Supplementary material for: Comparative Efficacy and Safety of PD-1/PD-L1 Inhibitors for Patients with Solid Tumors: A Systematic Review and Bayesian Network Meta-analysis
Source: J Cancer. 2021 Jan 1;12(4):1133–43. doi: 10.7150/jca.49325 (PMC7797652; doi:10.7150/jca.49325)
Supplement: Supplementary file 1 — Supplementary information, figures and table. [file jcav12p1133s1.pdf]

Supplementary materials

Search Strategies

Search strategy for Pubmed

(("Nivolumab"[Substance] OR “Nivolumab”[tiab] OR “Opdivo”[tiab] OR “MDX-1106”[tiab] OR “ONO-4538”[tiab] OR “BMS-936558”[tiab] OR “NIVO”[tiab]) OR ("Pembrolizumab"[Substance] OR “pembrolizumab” [tiab] OR “lambrolizumab”[tiab] OR “keytruda”[tiab] OR “MK-3475”[tiab] OR “SCH 900475”[tiab]) OR ("Atezolizumab"[Substance] OR "Atezolizumab"[tiab] OR "MPDL3280A"[tiab] OR "MSB0010718C"[tiab] OR "Tecentriq"[tiab] OR "RO5541267"[tiab] OR "RG7446"[tiab]) OR ("Durvalumab"[Substance] OR "Durvalumab "[tiab] OR "MEDI-4736"[tiab] OR "MEDI4736"[tiab]) OR ("Avelumab"[Substance] OR "Avelumab"[tiab] OR “Bavencio”[tiab] OR “MSB0010718C”[tiab]) OR ("checkpoint inhibitor"[All Field] OR " PD-1 "[All Fields] OR "PD-L1"[All Fields])) AND ("carcinoma"[Mesh] OR "cancer"[All Fields] OR "tumor"[tiab]) AND ("random allocation"[MeSH Terms] OR "randomized"[All Fields] OR "clinical trials as topic"[MeSH Terms] OR "trial"[All Fields] OR randomized controlled trial[All Field]) AND ("humans"[MeSH Terms])

Search strategy for Web of Knowledge

((“Nivolumab” OR “Opdivo” OR “MDX-1106” OR “ONO-4538” OR “BMS-936558” OR “NIVO”) OR (“pembrolizumab” OR “lambrolizumab” OR “keytruda” OR “MK-3475” OR “SCH 900475”) OR ("Atezolizumab" OR "MPDL3280A" OR "MSB0010718C" OR "Tecentriq" OR "RO5541267" OR "RG7446") OR ("Durvalumab " OR "MEDI-4736" OR "MEDI4736") OR ("Avelumab" OR “Bavencio” OR “MSB0010718C”) OR ("checkpoint inhibitor" OR " PD-1" OR "PD-L1")) AND ("carcinoma" OR "cancer" OR "tumor")
AND ("random allocation" OR "randomized" OR "clinical trials as topic" OR "trial" OR randomized controlled trial)

Supplementary Table S1. Jadad quality scores of the included trials

Trial Name	Randomization	Blinding	An account of all patients	Jadad score
KEYNOTE-002	2	0	1	3
CheckMate 037	2	0	1	3
CheckMate 066	2	2	1	5
CheckMate 067	2	2	1	5
CheckMate 069	2	2	1	5
KEYNOTE-006	2	0	1	3
CheckMate 238	2	2	1	5
KEYNOTE-054	2	0	1	3
KEYNOTE-010	2	0	1	3
KEYNOTE-024	2	0	1	3
POPLAR	2	0	1	3
OAK	2	0	1	3
PACIFIC	2	2	1	5
CheckMate 026	2	0	1	3
JAVELIN Lung 200	2	0	1	3
ARCTIC	1	0	1	2
CheckMate 057	2	0	1	3
KEYNOTE-021	2	0	1	3
KEYNOTE-189	2	2	1	5
IMpower 132	1	0	1	2
IMpower 150	2	0	1	3
CheckMate 017	2	0	1	3
KEYNOTE-407	2	2	1	5
IMpower 133	2	2	1	5
CheckMate 141	2	0	1	3
KEYNOTE-040	2	0	1	3
KEYNOTE-048	1	0	1	2
KEYNOTE-045	2	0	1	3
IMvigor211	2	0	1	3
CheckMate 025	2	0	1	3
JAVELIN Renal 101	1	0	1	2
ATTRACTION-2	2	2	1	5
KEYNOTE-061	2	0	1	3
JAVELIN Gastric 300	2	0	1	3
IMpassion 130	2	2	1	5

Supplementary Figure S1. Drug-based network meta-analysis in subsequent-line (A) and first-line (B) subgroups. Upper-triangle shows overall survival, and lower-triangle shows progression-free survival.

A

Control	**0.69(0.63-0.78)**	**0.75(0.68-0.83)**	**0.78(0.68-0.89)**	**0.63(0.46-0.86)**	0.97(0.80-1.18)
**0.79(0.63-0.99)**	Nivolumab	1.08(0.93-1.24)	1.12(0.93-1.31)	0.91(0.65-1.25)	**1.39(1.12-1.73)**
0.83(0.67-1.05)	1.05(0.77-1.45)	Pembrolizumab	1.03(0.87-1.22)	0.84(0.61-1.16)	**1.29(1.04-1.60)**
0.96(0.70-1.34)	1.22(0.82-1.80)	1.16(0.78-1.71)	Atezolizumab	0.81(0.58-1.14)	1.24(0.99-1.59)
0.71(0.40-1.27)	0.87(0.52-1.48)	0.85(0.45-1.58)	0.74(0.38-1.43)	Durvalumab	**1.54(1.07-2.21)**
1.36(0.90-2.10)	**1.72(1.07-2.79)**	**1.64(1.02-2.64)**	1.41(0.84-2.43)	1.92(0.95-3.98)	Avelumab

B

Control	**0.64(0.51-0.82)**	**0.64(0.53-0.79)**	0.78(0.60-1.01)	0.78(0.45-1.36)
**0.54(0.39-0.75)**	Nivolumab	1.00(0.73-1.37)	1.22(0.85-1.71)	1.22(0.66-2.21)
**0.66(0.50-0.88)**	1.22(0.79-1.87)	Pembrolizumab	1.21(0.87-1.68)	1.21(0.67-2.34)
**0.69(0.48-0.99)**	1.28(0.78-2.07)	1.04(0.66-1.65)	Atezolizumab	1.00(0.54-1.84)
0.69(0.33-1.43)	1.27(0.57-2.82)	1.04(0.48-2.27)	1.00(0.44-2.25)	Avelumab

Supplementary Figure S2. Drug-based network meta-analysis in placebo-controlled (A) and standard-of-care-controlled (SoC) (B) subgroups. Upper-triangle shows overall survival, and lower-triangle shows progression-free survival.

A

Control	**0.60(0.48-0.78)**	**0.63(0.51-0.80)**	**0.78(0.64-0.96)**	0.68(0.43-1.06)	0.78(0.50-1.22)
**0.45(0.33-0.61)**	Nivolumab	1.04(0.75-1.45)	1.29(0.93-1.75)	1.13(0.67-1.85)	1.29(0.76-2.13)
**0.61(0.48-0.77)**	1.34(0.92-1.97)	Pembrolizumab	1.24(0.91-1.64)	1.08(0.65-1.75)	1.23(0.74-2.02)
**0.69(0.53-0.90)**	**1.53(1.03-2.28)**	1.14(0.80-1.62)	Atezolizumab	0.87(0.53-1.42)	1.00(0.61-1.64)
**0.51(0.30-0.85)**	1.13(0.62-2.05)	0.84(0.48-1.47)	0.74(0.41-1.31)	Durvalumab	1.15(0.61-2.16)
0.69(0.41-1.16)	1.53(0.84-2.79)	1.14(0.64-2.02)	1.00(0.56-1.79)	1.35(0.65-2.81)	Avelumab

B

Control	**0.70(0.60-0.82)**	**0.71(0.61-0.83)**	**0.77(0.60-0.99)**	0.63(0.39-1.01)	0.98(0.72-1.36)
**0.74(0.59-0.93)**	Nivolumab	1.02(0.82-1.27)	1.10(0.82-1.47)	0.90(0.55-1.48)	1.41(0.99-2.01)
0.82(0.65-1.05)	1.11(0.80-1.54)	Pembrolizumab	1.08(0.81-1.44)	0.88(0.54-1.45)	1.38(0.97-1.96)
0.96(0.65-1.43)	1.31(0.83-2.05)	1.18(0.74-1.85)	Atezolizumab	0.82(0.48-1.40)	1.28(0.86-1.92)
0.71(0.35-1.43)	0.96(0.46-2.00)	0.87(0.41-1.80)	0.74(0.33-1.64)	Durvalumab	1.57(0.89-2.78)
1.38(0.84-2.30)	**1.87(1.08-3.25)**	1.68(0.97-2.95)	1.43(0.76-2.73)	1.95(0.82-4.63)	Avelumab

Supplementary Figure S3. Drug-based network meta-analysis in melanoma (A) and non-small-cell lung cancer (B) subroups. Upper-triangle shows overall survival, and lower-triangle shows progression-free survival.

A

Control	**0.63(0.46-0.89)**	0.74(0.44-1.23)
**0.51(0.39-0.70)**	Nivolumab	1.17(0.63-2.13)
**0.57(0.37-0.87)**	1.11(0.65-1.83)	Pembrolizumab

B

Control	**0.75(0.60-0.96)**	**0.58(0.48-0.71)**	**0.76(0.62-0.93)**	**0.65(0.48-0.90)**	0.90(0.60-1.34)
0.86(0.62-1.22)	Nivolumab	0.77(0.57-1.04)	1.01(0.73-1.37)	0.87(0.58-1.27)	1.19(0.74-1.88)
**0.60(0.46-0.79)**	0.70(0.45-1.08)	Pembrolizumab	1.31(0.98-1.72)	1.13(0.98-1.72)	1.55(0.99-2.39)
0.75(0.56-1.01)	0.87(0.55-1.36)	1.24(0.84-1.84)	Atezolizumab	0.86(0.59-1.25)	1.18(0.76-1.85)
**0.59(0.39-0.90)**	0.68(0.40-1.18)	0.97(0.60-1.62)	0.79(0.47-1.31)	Durvalumab	1.37(0.83-2.29)
1.16(0.64-2.11)	1.34(0.67-2.66)	1.92(1.00-3.70)	1.55(0.79-2.99)	1.97(0.94-4.06)	Avelumab

Supplementary Figure S4. (A) Sensitivity analysis of overall survival (upper triangle) and progression-free survival (lower triangle) according to the drug-based network meta-analysis. (Corresponding to Fig. 3A). (B) Sensitivity analysis of ≥Grade 3 treatment-related adverse events according to the drug-based network meta-analysis of placebo-controlled (upper triangle) or standard-of-care-controlled (SoC) trials (lower triangle) (Corresponding to Fig. 3B).

A

Control	**0.69(0.60-0.80)**	**0.69(0.62-0.78)**	**0.78(0.67-0.90)**	**0.65(0.48-0.89)**	0.92(0.72-1.16)
**0.76(0.61-0.97)**	Nivolumab	1.00(0.83-1.19)	1.12(0.91-1.37)	0.94(0.67-1.32)	**1.33(1.01-1.75)**
**0.74(0.61-0.90)**	0.97(0.72-1.31)	Pembrolizumab	1.12(0.93-1.35)	0.95(0.68-1.31)	**1.33(1.02-1.73)**
0.79(0.62-1.01)	1.03(0.73-1.45)	1.06(0.78-1.46)	Atezolizumab	0.84(0.60-1.19)	1.18(0.89-1.57)
**0.59(0.37-0.95)**	0.78(0.46-1.32)	0.80(0.48-1.33)	0.75(0.44-1.28)	Durvalumab	1.41(0.95-2.07)
1.06(0.72-1.60)	1.39(0.88-2.21)	1.43(0.93-2.23)	1.35(0.85-2.16)	1.79(0.97-3.32)	Avelumab

B

Placebo/SoC	2.37(0.68-8.29)	1.35(0.89-2.27)	1.17(0.71-1.94)	1.15(0.41-3.21)	1.00(0.37-2.73)
**0.36(0.26-0.50)**	Nivolumab	0.57(0.16-2.26)	0.49(0.13-1.91)	0.49(0.10-2.44)	0.42(0.09-2.09)
**0.39(0.28-0.53)**	1.07(0.68-1.72)	Pembrolizumab	0.87(0.41-1.65)	0.85(0.26-2.52)	0.74(0.23-2.12)
**0.37(0.22-0.60)**	1.02(0.57-1.85)	0.95(0.53-1.70)	Atezolizumab	0.98(0.31-3.07)	0.85(0.28-2.61)
0.67(0.29-1.58)	1.86(0.75-4.70)	1.74(0.70-4.33)	1.82(0.68-4.91)	Durvalumab	0.87(0.31-2.41)
**0.24(0.13-0.45)**	0.66(0.33-1.35)	0.61(0.30-1.24)	0.64(0.29-1.44)	0.35(0.12-1.02)	Avelumab

Supplementary Figure S5. Category-based network meta-analysis in subsequent-line (A) and first-line (B) subgroups. Upper-triangle shows overall survival, and lower-triangle shows progression-free survival.

A

Control	**0.73(0.67-0.78)**	**0.78(0.69-0.87)**
**0.81(0.70-0.95)**	PD-1 inhibitors	1.08(0.93-1.23)
0.95(0.75-1.20)	1.17(0.88-1.54)	PD-L1 inhibitors

B

Control	**0.64(0.55-0.76)**	**0.78(0.62-0.98)**
**0.80(0.68-0.94)**	PD-1 inhibitors	1.22(0.92-1.60)
0.96(0.75-1.25)	1.21(0.89-1.64)	PD-L1 inhibitors

Supplementary Figure S6. Category-based network meta-analysis in placebo-controlled (A) and standard-of-care-controlled (SoC) (B) subgroups. Upper-triangle shows overall survival, and lower-triangle shows progression-free survival.

A

Control	**0.62(0.54-0.72)**	**0.77(0.65-0.90)**
**0.54(0.44-0.66)**	PD-1 inhibitors	1.24(0.99-1.53)
**0.66(0.51-0.84)**	1.21(0.88-1.67)	PD-L1 inhibitors

B

Control	**0.71(0.64-0.79)**	**0.81(0.69-0.95)**
**0.80(0.68-0.94)**	PD-1 inhibitors	1.15(0.94-1.38)
0.96(0.75-1.25)	1.21(0.89-1.64)	PD-L1 inhibitors

Supplementary Figure S7. Category-based network meta-analysis in non-small-cell lung cancer (A), urinary system cancer (B) and Gastric or gastro-esophageal junction cancer (C) subgroups. Upper-triangle shows overall survival, and lower-triangle shows progression-free survival.

A

Control	**0.65(0.55-0.77)**	**0.75(0.63-0.89)**
**0.69(0.55-0.87)**	PD-1 inhibitors	1.17(0.91-1.46)
**0.75(0.59-0.97)**	1.09(0.77-1.53)	PD-L1 inhibitors

B

Control	0.73(0.49-1.08)	0.83(0.55-1.21)
0.92(0.47-1.86)	PD-1 inhibitors	1.14(0.64-1.94)
0.79(0.41-1.68)	0.86(0.34-2.40)	PD-L1 inhibitors

C

Control	0.71(0.25-2.07)	1.10(0.24-4.98)
0.86(0.21-3.58)	PD-1 inhibitors	1.55(0.24-9.70)
1.73(0.23-13.1)	2.01(0.17-24.2)	PD-L1 inhibitors

Supplementary Figure S8. (A) Sensitivity analysis of overall survival (upper triangle) and progression-free survival (lower triangle) according to the category-based network meta-analysis. (Corresponding to Fig. 5A). (B) Sensitivity analysis of ≥Grade 3 treatment-related adverse events according to the category -based network meta-analysis of placebo-controlled (upper triangle) or standard-of-care-controlled (SoC) trials (lower triangle) (Corresponding to Fig. 5C).

A

Control	**0.69(0.63-0.76)**	**0.75(0.66-0.86)**
**0.78(0.67-0.89)**	PD-1 inhibitors	1.09(0.92-1.28)
**0.75(0.61-0.93)**	0.97(0.76-1.25)	PD-L1 inhibitors

B

Placebo/SoC	**2.46(1.95-3.12)**	**2.82(1.90-4.20)**
**0.37(0.29-0.47)**	PD-1 inhibitors	0.71(0.43-1.10)
**0.36(0.25-0.51)**	0.95(0.61-1.48)	PD-L1 inhibitors
